# Bibliometric and visual analysis of global crc circular RNA research 2015-2023

**DOI:** 10.3389/fimmu.2025.1580405

**Published:** 2025-06-18

**Authors:** Jian Feng Bin, Long Fei Chen, Yan Wang, Hua Ge, Wei Chen

**Affiliations:** ^1^ Department of Gastrointestinal Surgery, The Third Affiliated Hospital of Zunyi Medical University (Zunyi First People's Hospital), Zunyi, China; ^2^ Hepatobiliary Surgery Department, The Third Affiliated Hospital of Zunyi Medical University (The First People’s Hospital of Zunyi), Zunyi, China; ^3^ Department of Laboratory Medicine, The Third Affiliated Hospital of Zunyi Medical University (The First People’s Hospital of Zunyi), Zunyi, China; ^4^ Department of Gastrointestinal Surgery, The Third Affiliated Hospital of Zunyi Medical University (The First People's Hospital of Zunyi), Zunyi, China

**Keywords:** circular RNA, CRC, review, bibliometric analysis, visualisation analysis, Citespace, VOSviewer

## Abstract

**Background:**

Colorectal cancer (CRC) is the third highest malignant tumor in the world in terms of incidence rate, accounting for about 10% of all cancer cases and the second leading cause of cancer related deaths. Timely diagnosis and effective treatment are key to significantly improving the survival rate of CRC patients. Various factors including gender, environmental factors, lifestyle choices, and genetic predisposition are all causes of the onset of CRC. Circular RNA(circRNA) mainly exists in cancer cells and tissues, solid tumors, peripheral blood, exosomes, and body fluids (such as serum, plasma, and saliva). Due to their resistance to degradation and presence in body fluids, CircRNA is non-invasive and an ideal candidate for liquid biopsy, thus having high diagnostic potential. Current research has found that circRNA can regulate the proliferation, migration, invasion, and apoptosis of CRC cells.

**Methods:**

This article aims to comprehensively evaluate the current status and trends of circRNA research in the field of CRC through bibliometric analysis. Intended to use software tools such as VOSviewer, CiteSpace, Microsoft Excel, and R language package to illustrate research progress and focus, in order to outline development trends and future research directions.

**Results:**

This study used specific inclusion and exclusion criteria to investigate 1048 papers published before December 31, 2023 in the core database of the World Council for Scientific Knowledge. Our research findings indicate that China has the highest number of published papers in this field, with Nanjing Medical University and Kiavash Hushmandi considered the most influential institutions and authors. The Journal ofOncology Frontiers is the journal with the most published papers, while the article "Circular RNA: The New Star of Non coding RNA" is the journal with the most published papers. In addition, terms such as "signaling pathway", "promoting metastasis", "promoting proliferation", and "drug resistance" have become important focuses in this research field.

**Conclusions:**

This study indicates that the focus between circRNA and CRC has shifted from studying the biological characteristics of circRNA to studying the regulation of circRNA molecular mechanisms. Future research should emphasize the potential use of circRNA as a biomarker for the diagnosis, prognosis, and treatment of CRC.

## 1 Introduction

CRC incidence and mortality have risen worldwide in recent decades (1). Data indicate that the worldwide burden of CRC may surge by 60% by 2030, exceeding 2 million new cases and over 1 million deaths (2, 3). Significant regional disparities exist in CRC incidence, with declining rates in Europe and the US recently, whereas Southeast Asia has seen a gradual rise (4). The onset of CRC is subtle, with early symptoms often not apparent. At diagnosis, 30-50% of patients present with lymph node or distant metastasis, resulting in missed optimal treatment windows, a 5-year survival rate of approximately 14%, and poor regression post-chemotherapy (5). Early prevention and diagnosis are therefore crucial. Currently, genetics, biology, and chromosomal factors are closely linked to CRC, with survival also influenced by biological differences in molecular subtypes, typically resulting in a 2–3 year survival period (6).

Genomic sequencing of tissues from cancer patients has shown that CRC patients have abnormal expression of (circRNA) in blood, cells, tissues and exosomes. Certain circRNAs are over- or under-expressed in tumour tissues, showing sensitivity and diagnostic potential for cancer progression (7). Dysregulation of circRNAs can also be targeted to affect tumour tissues’ sensitivity to radiotherapy. For as whether it can be used as a new biomarker, it needs to be continuously explored and validated (8). Researchers discovered that circRNAs predominantly exist in mammalian cells, primarily due to their absence of a 5’ cap and poly-A tail, forming a closed-loop structure. Most circRNAs rely on “reverse splicing” for their function (9), initially thought to result from splicing errors and deemed inefficient, hence classified as low-functioning RNAs (10). Subsequent exploration of over 20,000 circRNAs through high-throughput sequencing revealed that circRNAs are a type of non-coding RNAs predominantly present in eukaryotes. They exhibit long half-lives and resistance to nuclease degradation due to their closed-loop structure (11). Increasing studies have highlighted the unique characteristics of circRNAs, including high stability, resistance to nucleic acid exonucleases, and presence in body fluids (abundance, stability) (12). With a diversity exceeding 20,000 identified types, circRNAs show conservation and stable sequence evolution in mammals. They exhibit specific expression patterns across different time periods and tissues (13). Given this context, further investigation into circRNAs’ role in CRC is crucial (7). circRNAs can regulate tumour cell proliferation, invasion, and metastasis, either promoting or inhibiting cancer development. Thus, circRNAs are seen as a new generation of markers and a focus for targeted drug-resistant therapies. Many researchers have studied the impact of circRNAs on CRC progression, development of CRC, which also indicates that this field still has significant research potential (14).

In recent years, bibliometric analysis has been widely used to assess the impact of articles and identify research hotspots, using both quantitative and qualitative methods to collect large amounts of bibliometric data from core databases, aiming to reveal research hotspots and trends by acquiring, processing and analysing data on countries, institutions, authors, journals, articles and keywords in the research area. This helps to summarise the body of knowledge and research trends (15). Bibliometrics measures the output and contribution of an author, institution or country by analysing the number of published articles, journal impact factor and number of citations (16).

Increasing literature confirms the significant potential of circRNA in the early diagnosis and prognosis of CRC, circRNA is crucial in CRC development (17). As a potential biomarker, it has garnered attention in studying gastrointestinal tumors (8). However, there are few quantitative studies on research hotspots and directions in this field. To address this gap, we will utilize bibliometric and visualization analyses to collect and forecast the current status and progress of global research on CRC and circRNA. We will use big data to visualize recent research trends, providing a comprehensive overview of current knowledge and understanding of circRNA in diagnosing and treating CRC (18).

## 2 Methods

### 2.1 Collection of literature

**Figure 1:**
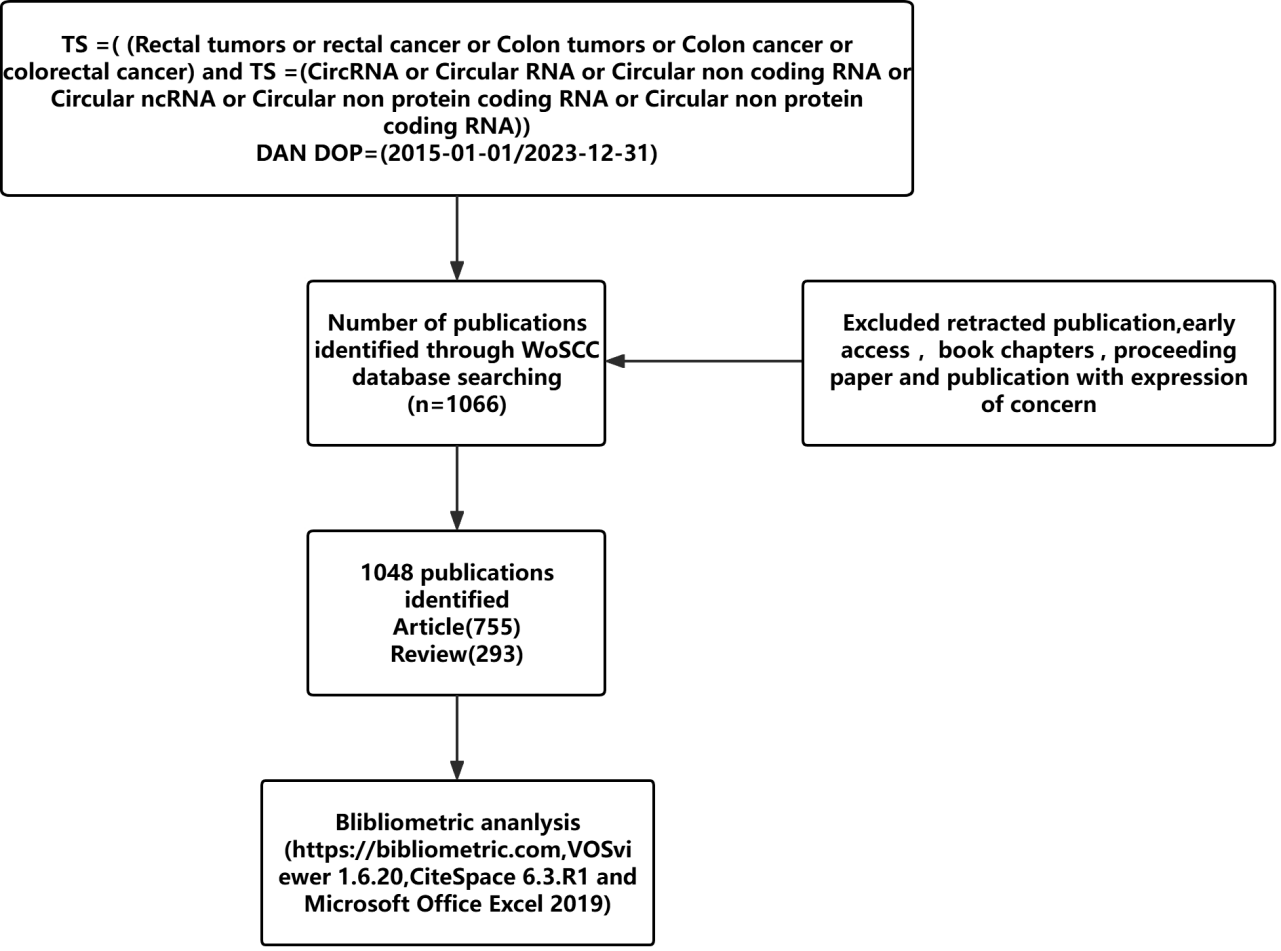
Flowchart of the inclusion and exclusion process for the CircRNA and CRC Study.

Data from 1 January 2015 to 31 December 2023 were collected using the WoSCC database. The search strategy was: TS =( Rectal tumors or rectal cancer or Colon tumors or Colon cancer or colorectal cancer) and TS = (CircRNA or Circular RNA or Circular non coding RNA or Circular ncRNA or Circular non protein coding RNA or Circular non protein coding RNA). The final search was performed on 1 July 2024, with inclusion criteria: 1) language: English, Chinese; 2) publication year: 2015-2023. A total of 1048 publications were included. Exclusion criteria were document types such as ‘retracted publication, early access, books chapters, proceeding paper and publication with expression of concern’. Full records and cited references for all qualifying publications were exported and downloaded as plain text, tab-delimited files. The data included the number of papers and citations, titles, authors, year of publication, country, institution, journal, keywords, abstracts, and references for bibliometric analyses. To ensure accurate interpretation of the results, the language of publication was restricted to English. The search process can be seen in **Figure 1**. We also use the correlation between national GDP and different bibliometrics to provide a more comprehensive evaluation of research output efficiency, and the information on Gross Domestic Product (GDP) comes from the World Bank, as shown in **Table 1**.

**Table 1:** Top 14 countries that contributed to the publication CircRNA in CRC (Taiwan incorporated into the People’s Republic of China).

Country	Documents	Citations	Total link strength	GDP (in trillions of dollars)
peoples r china	885	29968	55	130.965
iran	64	1266	52	3.53441
usa	53	3755	44	197.671
germany	15	568	16	35.7114
italy	15	571	3	18.3129
canada	12	453	16	16.1699
india	10	186	8	25.3515
turkey	10	461	17	7.69854
iraq	9	64	15	1.90981
singapore	9	409	14	3.50761
australia	8	421	9	13.0083
england	8	437	6	26.3549=
malaysia	8	472	2	3.16424
pakistan	8	103	13	2.99158

### 2.2 Visualisation and analysis

Data visualization was conducted using VOSviewer 1.6.20 and CiteSpace 6.3.R1. VOSviewer, a free JAVA-based software developed by the Centre for Scientific and Technological Research at the University of Leiden, Netherlands, in 2007, is designed for literature data analysis and the visualization of constructed network models, focusing on the visualization of literature knowledge (19). CiteSpace, developed by the School of Information Science and Technology at the University of Lexelles (20), is an information visualization tool that enables in-depth mining and analysis of literature and data across various domains. We utilized BIBLIOMETRIC (https://bibliometric.com) for basic literature analysis. These tools were employed to analyze the data, and the results were exported and summarized in a table of bibliometric parameters, including the number and year of publications, total citations, titles, country and institution, authors, journals, and keywords.

## 3 Results

### 3.1 Trends in publications and citations

**Figure 2:**
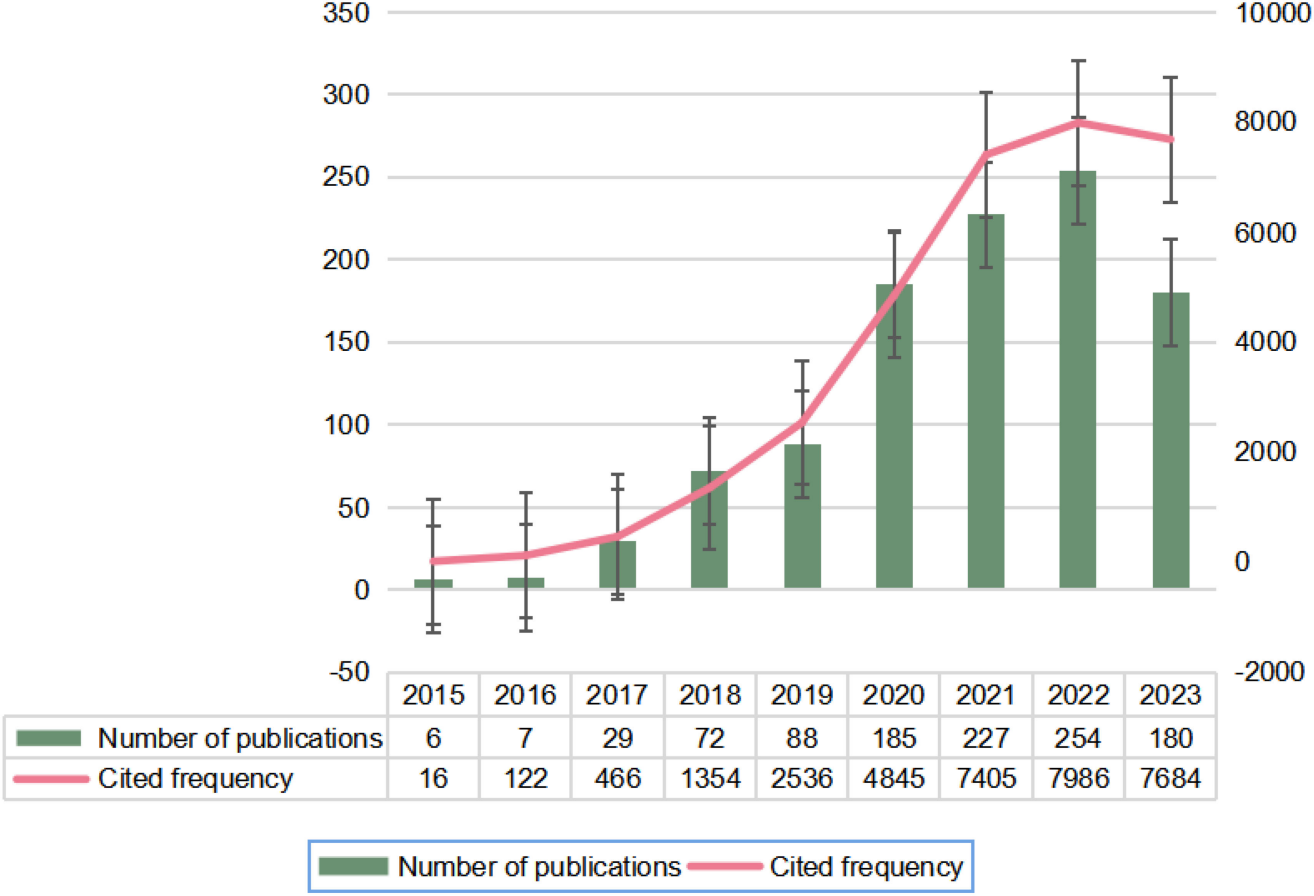
Annual publication and citation counts for global circRNA-associated CRC research, 2015-01-01-2023-12-31.

Following our search strategy, a total of 1048 publications, including ‘articles’ (n=755) and ‘reviews’ (n=293), were included after excluding those that did not meet the inclusion criteria. **Figure 2** illustrates the global publication and total citation trends for circRNA-related CRC research from 1 January 2015 to 31 December 2023. There has been a general upward trend in publications over the past nine years, starting with single-digit publications in 2015–2016 when research hotspots were first explored, and then increasing to 29 publications in 2017 as interest grew among researchers. Until 2022, the number of publications showed a significant year-on-year increase, peaking in 2022. The most notable rise occurred from 2019 to 2020. However, in 2023, the number of publications began to decline. The fluctuation in the number of citations corresponds to the trend in the number of publications. Despite the decline, the average number of publications over the past three years has remained high at 222. The overlap in publication and citation trends indicates that the field continues to receive significant attention from researchers.

### 3.2 Popular articles

The top 20 most cited articles in circRNA-related CRC research were identified based on their citation counts on Web of Science (**Table 2**), ranging from 1378 (“Circular RNA: A new star of noncoding RNAs”) to 254 (“cir-ITCH Plays an Inhibitory Role in CRC by Regulating the Wnt/β-Catenin Pathway”). Most of these highly cited articles investigate circRNA as a novel, stable biomarker for cancer, serving as a target or vector for cancer therapy, and explore its biological and molecular mechanisms. Early studies outlined the biological origin and characteristics of circRNAs, gradually linking them with specific diseases, exploring their physiopathological mechanisms, and consistently highlighting their significance. Recent research has shifted towards basic experiments and biological analyses of circRNAs, confirming their significant potential for disease diagnosis and treatment, targeted drug therapy, and understanding drug resistance mechanisms. The potential of circRNAs in diagnosing and treating diseases, targeted drug therapy, and drug resistance mechanisms is vast, aiming to enhance early cancer intervention and patients’ quality of life. The top-listed articles are published in journals known for swiftly disseminating extensive research in cancer studies (e.g., Cancer Letters), with recent research hotspots ranking highly. For instance, Wang, Xinyi’s (2020) study was featured in a leading international journal of molecular oncology. These studies emphasized the fundamental properties of circRNAs, laying a robust research foundation for their application in CRC.

**Table 2:** Top 20 publications based on citations.

Title	Authors	Source Title	Publication Year	Total Citations
Circular RNA: A new star of noncoding RNAs	Qu, Shibin	CANCER LETTERS	2015	1378
Circular RNA and its mechanisms in disease: From the bench to the clinic	Han, Bing; Chao	PHARMACOLOGY & THERAPEUTICS	2018	618
CircRNA: a novel type of biomarker for cancer	Zhang, He-da	BREAST CANCER	2018	617
Correlation of circular RNA abundance with proliferation - exemplified with colorectal and ovarian cancer, idiopathic lung fibrosis, and normal human tissues	Bachmayr-Heyda, Anna	SCIENTIFIC REPORTS	2015	592
Noncoding Effects of Circular RNA CCDC66 Promote Colon Cancer Growth and Metastasis	Hsiao, Kuei-Yang	CANCER RESEARCH	2017	519
CircHIPK3 promotes CRC growth and metastasis by sponging miR-7	Zeng, Kaixuan	CELL DEATH & DISEASE	2018	479
N6-methyladenosine modification of circNSUN2 facilitates cytoplasmic export and stabilizes HMGA2 to promote colorectal liver metastasis	Chen, Ri-Xin	NATURE COMMUNICATIONS	2019	429
The interplay between m6A RNA methylation and noncoding RNA in cancer	Ma, Shuai	JOURNAL OF HEMATOLOGY & ONCOLOGY	2019	381
Emerging roles of circRNA_001569 targeting miR-145 in the proliferation and invasion of CRC	Xie, Huijun	ONCOTARGET	2016	379
Circular RNA ciRS-7-A Promising Prognostic Biomarker and a Potential Therapeutic Target in CRC	Weng, Wenhao	CLINICAL CANCER RESEARCH	2017	365
Circular RNA participates in the carcinogenesis and the malignant behavior of cancer	Zhao, Zhen-Jun	RNA BIOLOGY	2017	347
Exosome-delivered circRNA promotes glycolysis to induce chemoresistance through the miR-122-PKM2 axis in CRC	Wang, Xinyi	MOLECULAR ONCOLOGY	2020	337
Circular RNA circ-ITCH inhibits bladder cancer progression by sponging miR-17/miR-224 and regulating p21, PTEN expression	Yang, Chengdi	MOLECULAR CANCER	2018	325
Circular RNAs in Cancer	Bach, Duc-Hiep	MOLECULAR THERAPY-NUCLEIC ACIDS	2019	322
A novel protein encoded by a circular RNA circPPP1R12A promotes tumor pathogenesis and metastasis of colon cancer via Hippo-YAP signaling	Zheng, Xiao	MOLECULAR CANCER	2019	297
CircRNAs and cancer: Biomarkers and master regulators	Arnaiz, Esther	SEMINARS IN CANCER BIOLOGY	2019	290
Exosomal circPACRGL promotes progression of CRC via the miR-142-3p/miR-506-3p-TGF-β1 axis	Shang, Anquan	MOLECULAR CANCER	2020	282
Circular RNA: a novel biomarker and therapeutic target for human cancers	Lei, Bo	INTERNATIONAL JOURNAL OF MEDICAL SCIENCES	2019	265
Circular RNAs are down-regulated in KRAS mutant colon cancer cells and can be transferred to exosomes	Dou, Yongchao	SCIENTIFIC REPORTS	2016	261
cir-ITCH Plays an Inhibitory Role in CRC by Regulating the Wnt/β-Catenin Pathway	Huang, Guanli	PLOS ONE	2015	254

### 3.3 Country analysis of publications

**Figure 3:**
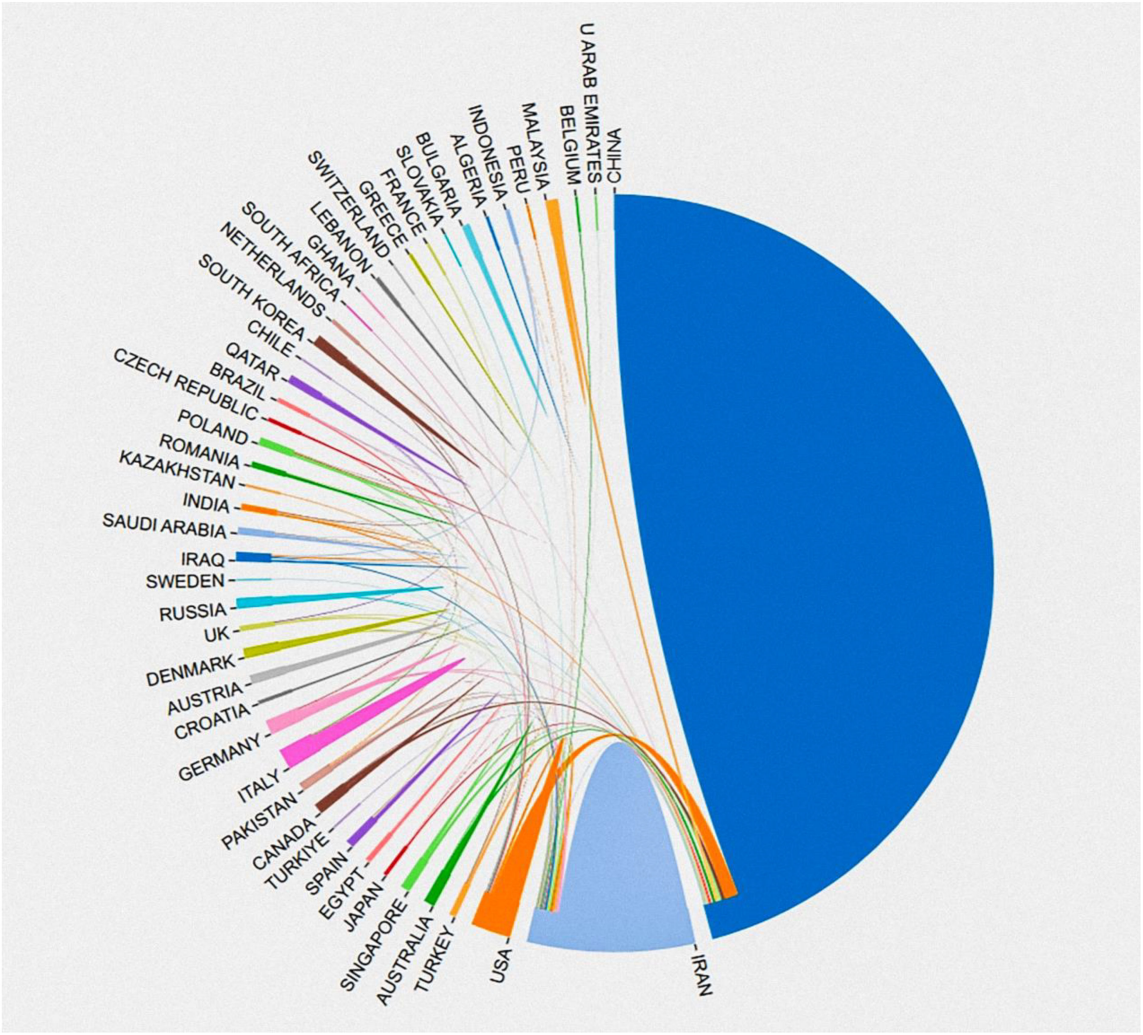
Visualisation map of countries with international cooperation.

From 2015 to 2023, a total of 49 countries contributed to research on the role of circRNAs in CRC. **Table 1** lists the 14 most productive countries in this field, and displayed the corresponding country’s Gross Domestic Product (GDP). According to the data distribution, the majority of publications were from China (885 articles, approximately 84.4%, including Taiwan). Iran (64 articles, about 6.1%) and the United States (53 articles, about 5.05%) ranked second and third, respectively. The number of articles published in China was more than ten times that of these countries, highlighting a significant disparity. Similarly, Chinese studies received the highest number of citations, totaling 29,968, which is several times more than Iran (1266 citations) and the USA (3755 citations). The remaining countries did not surpass 1,000 citations and were thus not specified. This indicates that the majority of research in this area over the past nine years has been concentrated in China, significantly impacting the field. In terms of the corresponding GDP, China, which ranks first in terms of the number of publications, has a GDP of ($130.965 trillion), while the United States, which ranks third, has a GDP of ($197.671 trillion), and even more concerning is that iran, which ranks second, has a GDP of only ($3.53441 trillion), which indirectly suggests that the GDP does not show a positive correlation with research productivity. **Figure 3** shows the collaboration between different countries, with China having the highest proportion of multi-country publications and a high rate of cooperation with other countries. The US has comparatively less interaction with other nations.

### 3.4 Analysis of the publications organization

**Figure 4:**
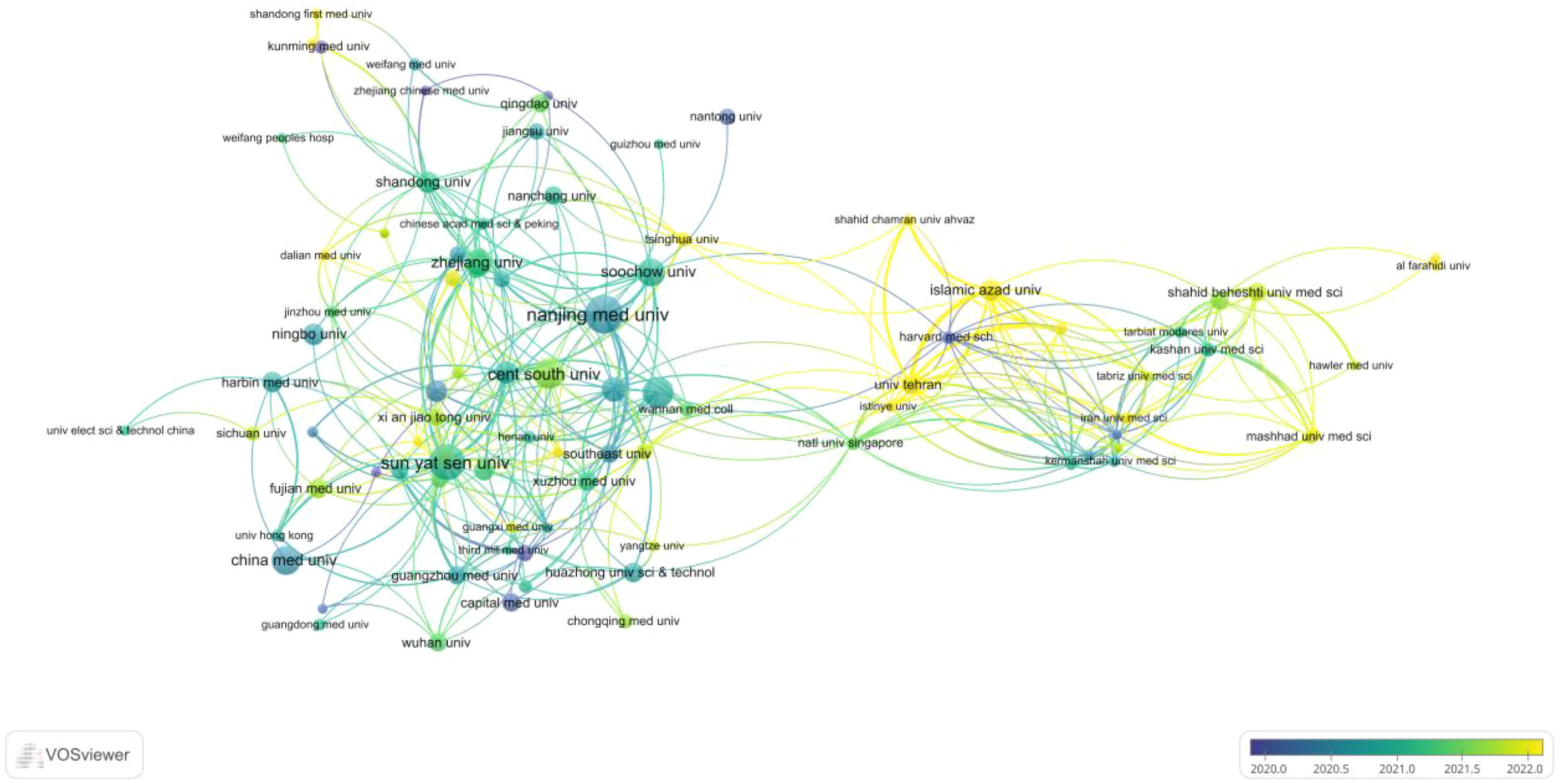
Linkages between different research organisations and for the study of CircRNA associated with CRC VOSviewer visual time network diagram.

A total of 1006 institutions contributed to publications on circRNA-related CRC research. The 35 collaborating institutions, selected from these 1006, were visualized and analyzed using VOSviewer to create a table. The top 35 most productive institutions, each with at least 16 publications, are shown in **Table 3**. As shown in **Table 3**, 27 of the top 35 institutions are from China. This highlights the need to strengthen transnational collaborations, as most cooperating institutions are domestic. The institution with the highest number of publications is Nanjing Medical University (59), which also has the closest linkage strength with other institutions, totaling 663. This is followed by Sun Yat-sen University (51 publications; total linkage strength of 509), Central South University (38 publications; total linkage strength of 431), and Zhengzhou University (37 publications; total linkage strength of 325). The co-authorship network visualization (**Figure 4**) clearly shows that a large proportion of publishing institutions in this field are based in China. The earliest institutions initiating this research also originated in China. However, in recent years, new research institutions have mostly been from abroad, indicating that this research field is gradually receiving global attention.

**Table 3:** Top 35 institutions that contributed to the publication CircRNA in CRC.

Organization	Documents	Citations	Total link strength
nanjing med univ	59	3076	663
sun yat sen univ	51	2394	509
cent south univ	38	783	431
zhengzhou univ	37	1693	325
china med univ	33	730	285
soochow univ	31	1295	360
zhejiang univ	28	791	272
fudan univ	25	1366	353
southern med univ	21	830	222
shanghai jiao tong univ	19	1155	178
ningbo univ	19	500	175
islamic azad univ	18	431	182
harbin med univ	17	475	135
anhui med univ	16	561	128
xuzhou med univ	15	1116	180
huazhong univ sci & technol	15	732	145
jilin univ	15	484	133
univ tehran	15	465	167
capital med univ	14	734	145
southeast univ	13	2192	299
guangzhou med univ	13	818	137
wenzhou med univ	12	721	130
chinese acad sci	11	688	110
tongji univ	10	795	168
univ hong kong	6	557	77
univ texas md anderson canc ctr	4	726	48
med univ vienna	3	594	63
tianjin med univ canc inst & hosp	3	486	75
fourth mil med univ	2	1379	82
baylor coll med	2	615	89
natl cheng kung univ	2	522	94
shanghai inst digest dis	2	465	67
ludwig boltzmann cluster translat oncol	1	592	63
state key lab oncol south china	1	427	52
xentech sas	1	427	52

### 3.5 Analysis of authors of publications

This study surveyed 5582 authors, with the top 10 presented in **Table 4**. Each of these authors has published at least 8 articles. The top three authors each published 12 articles. Hushmandi Kiavash (Baqiyatallah University of Medical Sciences) has 12 publications with 443 citations and a total link strength of 27; Liu Wei (Jilin University) has 12 publications with 397 citations and a total link strength of 11; Zhang Yi (Xuzhou Medical University) has 12 publications with 390 citations and a total link strength of 17. Additionally, Chen (Zhengzhou University) ranked 8th with 8 articles and 1105 citations, and a total linkage strength of 6, having the highest number of total citations despite fewer publications, indicating high impact. In conclusion, their research has significantly impacted the field of circRNA in CRC.

**Table 4:** Top 10 authors with the most publications in CRC and circRNA research.

Author	Documents	Citations	Total link strength
hushmandi, kiavash	12	433	27
liu, wei	12	397	11
zhang, yi	12	390	17
zhang, wei	11	673	0
zhang, lei	11	296	0
zarrabi, ali	9	470	20
hashemi, mehrdad	9	108	19
chen, chen	8	1105	6
liu, yang	8	222	5
taheriazam, afshin	8	106	19

### 3.6 Analysis of publication journals and strongest citation bursts

**Figure 5:**
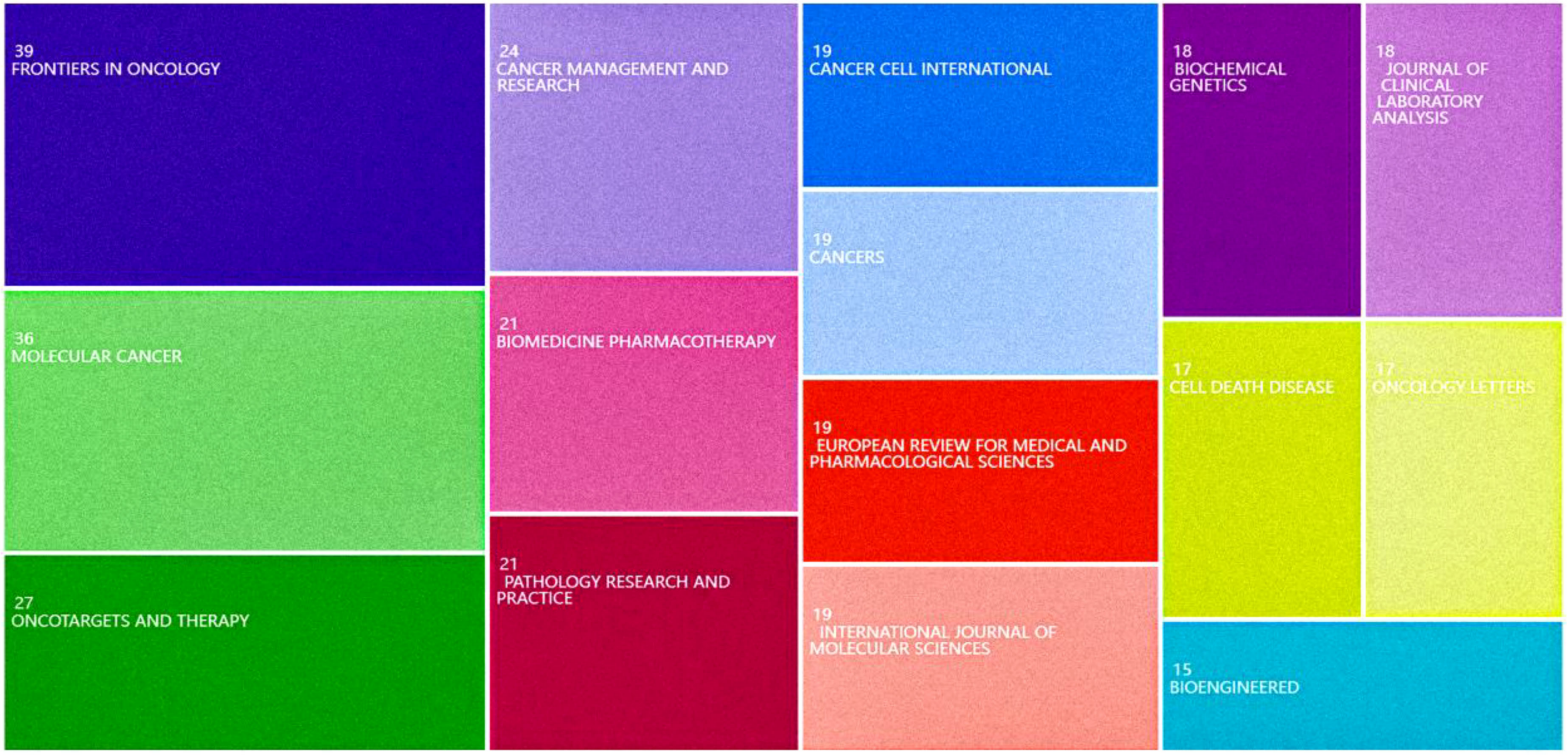
Dendrogram of the top 15 journals with the highest number of research publications related to CircRNA and CRC, 2015-2023.

**Figure 6:**
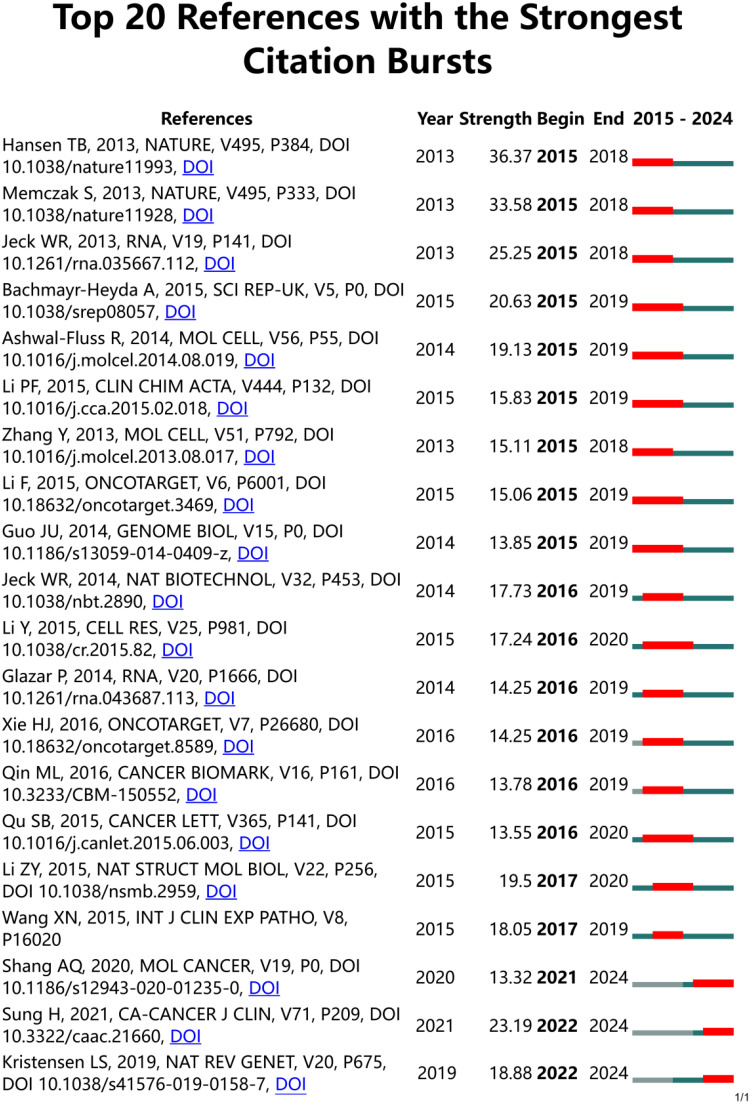
The top 20 references with the strongest citation bursts between 2015 and 2023 were involved in CircRNA in CRC, as determined by CiteSpace. red lines represent burst times.

The top ten most productive journals contributed 328 articles, as in **Figure 5** representing about 31.39% of the total publications. Out of 303 academic journals, the top three in terms of number of publications were *Frontiers In Oncology* (39 articles), *Molecular Cancer* (36 articles), and *Oncotargets and Therapy* (27 articles). The impact factor (IF) of a journal is a key indicator used to measure its importance. The IF is universal, objective, and reliable, making it a crucial metric for evaluating articles in the research community, both for the quality of the research and the impact of the findings (21). *Molecular Cancer* had the highest impact factor (IF 2022 = 37.3), followed by *Frontiers In Oncology* (IF 2023 = 3.5) and *Oncotargets and Therapy* (IF 2023 = 2.7). The top 20 references with the strongest citation burst are shown in **Figure 6**. These citations are distributed over specific time periods, reflecting the research hotspots of those times. The first citation burst occurred between 2013 and 2018, with Hansen TB et al. (2013) having the highest burst intensity (36.37), followed by Memczak S et al. (2013) (33.58 intensity) and Jeck WR et al. (2013) (25.25 intensity), indicating a high level of interest. The latest citation bursts, with Sung H et al. (2021) (23.19 intensity) and Kristensen LS et al. (2019) (18.88 intensity), have been receiving increasing attention in recent years.

### 3.7 Analysis of co-cited references

**Figure 7:**
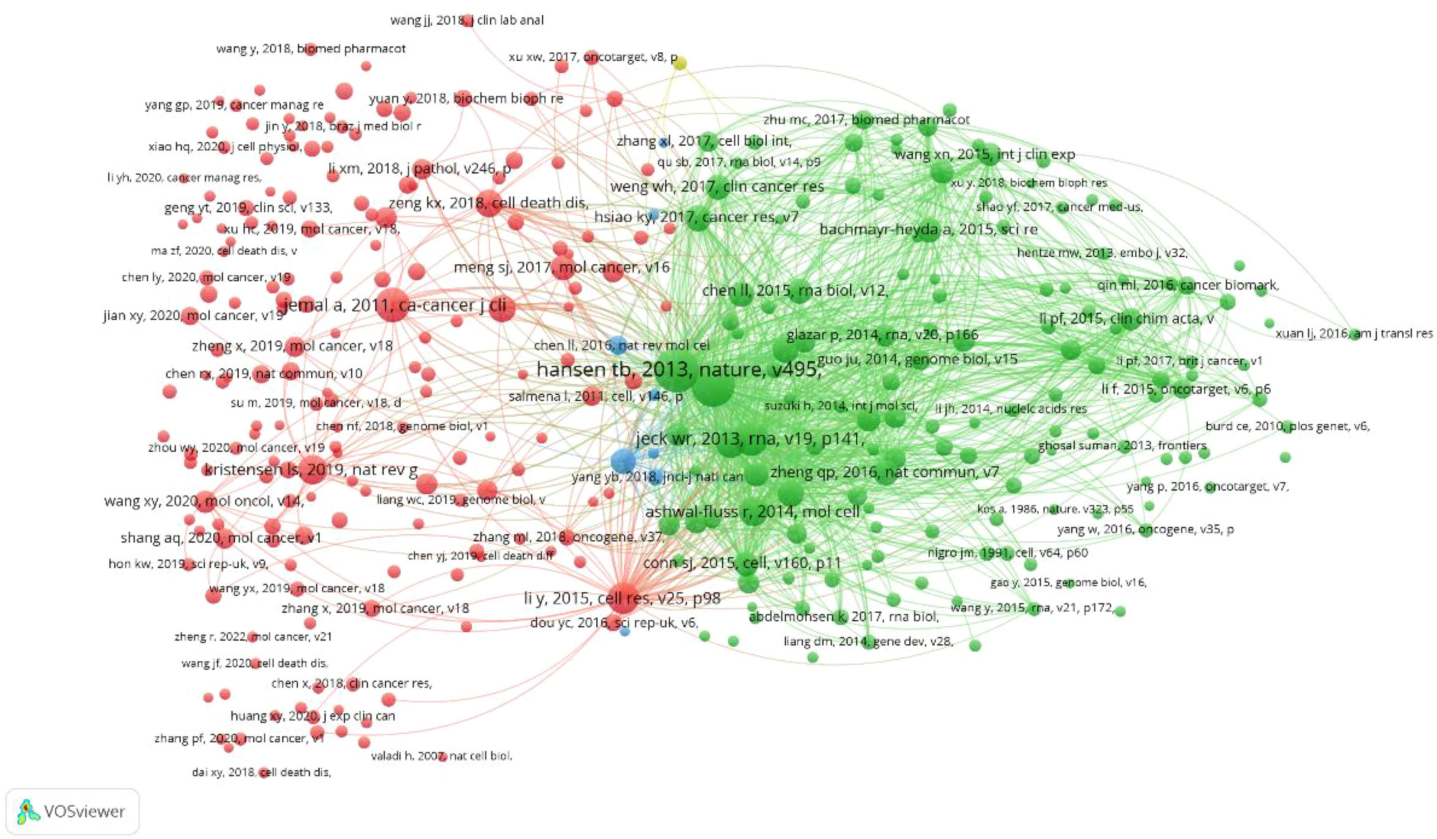
CRC on CircRNA research VOSviewer visual network common references: Node size reflects the co citation strength and citation frequency of the literature, node color clustering represents a research field, and node position proximity represents literature topic relevance.

The cited references serve as the theoretical foundation and knowledge framework for the entire research topic. If a paper cites two references simultaneously, their contents may be relevant. The more times they are referenced together, the stronger the correlation. Therefore, statistical analysis of commonly cited references has guiding significance. As shown in the figure, out of a total of 41533 references, we used the minimum citation count (20 times) of the cited references. A total of 328 cited references are shown in **Figure 7**, where the size of nodes reflects the co citation strength and citation frequency of the references. Node color clustering represents a research field, and node position represents whether the topic is relevant or not. We further displayed the first 22 co cited references, with each reference cited 100 times, as shown in **Table 5**: The first three research directions mainly explore the biological characteristics related to circRNA. The most commonly cited references mainly reveal the miRNA sponge function of circRNA through experiments, providing a new perspective for studying circRNA as a competitive endogenous RNA regulatory target gene (Thomas B Hansen et al., 2013). The second ranked reference sequenced and calculated circRNA as a posttranscriptional regulator, indicating that circRNA has important regulatory potential (Sebastian Memczak et al., 2013). The third reference uses bioinformatics methods to reveal the rich, stable, and conserved functions of circRNAs, which can serve as novel regulatory molecules for gene expression. (William R Jeck et al., 2013).

**Table 5:** The top 22 co cited references for the number of studies related to CircRNA in CRC.

Cited reference	Citations	Total link strength
hansen tb, 2013, nature, v495, p384, doi 10.1038/nature11993	348	1737
memczak s, 2013, nature, v495, p333, doi 10.1038/nature11928	344	1664
jeck wr, 2013, rna, v19, p141, doi 10.1261/rna.035667.112	219	1364
jemal a, 2011, ca-cancer j clin, v61, p134, doi [10.3322/caac.20115 10.3322/caac.20107 10.3322/caac.21492]	213	657
li y, 2015, cell res, v25, p981, doi 10.1038/cr.2015.82	173	954
li zy, 2015, nat struct mol biol, v22, p256, doi 10.1038/nsmb.2959	171	1169
ashwal-fluss r, 2014, mol cell, v56, p55, doi 10.1016/j.molcel.2014.08.019	159	1063
kristensen ls, 2019, nat rev genet, v20, p675, doi 10.1038/s41576-019-0158-7	156	490
jemal a, 2009, ca-cancer j clin, v59, p225, doi [10.3322/caac.21601 10.3322/caac.20006 10.3322/caac.21332 10.3322/caac.21654 10.3322/caac.20073 10.3322/caac.21254 10.3322/caac.21387 10.3322/caac.21551]	138	518
zeng kx, 2018, cell death dis, v9, doi 10.1038/s41419-018-0454-8	135	602
zhang y, 2013, mol cell, v51, p792, doi 10.1016/j.molcel.2013.08.017	129	949
hsiao ky, 2017, cancer res, v77, p2339, doi 10.1158/0008-5472.can-16-1883	126	780
jeck wr, 2014, nat biotechnol, v32, p453, doi 10.1038/nbt.2890	123	728
chen ll, 2015, rna biol, v12, p381, doi 10.1080/15476286.2015.1020271	119	649
meng sj, 2017, mol cancer, v16, doi 10.1186/s12943-017-0663-2	119	432
weng wh, 2017, clin cancer res, v23, p3918, doi 10.1158/1078-0432.ccr-16-2541	119	726
zheng qp, 2016, nat commun, v7, doi 10.1038/ncomms11215	115	734
sanger hl, 1976, p natl acad sci usa, v73, p3852, doi 10.1073/pnas.73.11.3852	113	743
qu sb, 2015, cancer lett, v365, p141, doi 10.1016/j.canlet.2015.06.003	112	559
conn sj, 2015, cell, v160, p1125, doi 10.1016/j.cell.2015.02.014	111	793
du ww, 2016, nucleic acids res, v44, p2846, doi 10.1093/nar/gkw027	108	775
bachmayr-heyda a, 2015, sci rep-uk, v5, doi 10.1038/srep08057	107	696

### 3.8 Keyword analysis of publications

**Figure 8:**
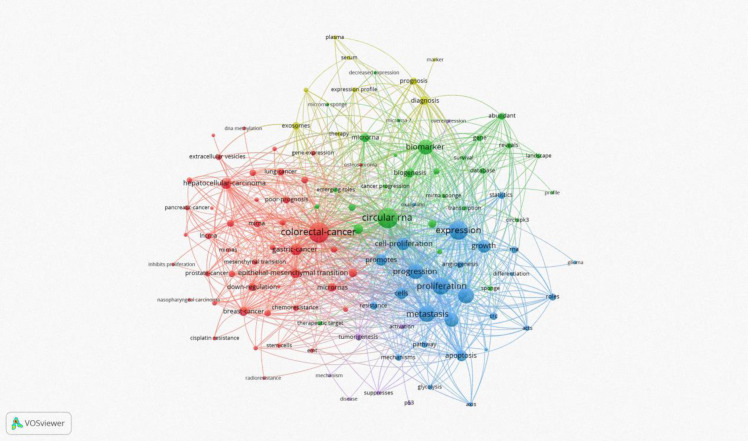
CRC on CircRNA research keywords for the VOSviewer visual network: keyword mapping. All keywords were divided into five groups and represented by different colors. The larger the circle, the higher the frequency.

**Figure 9:**
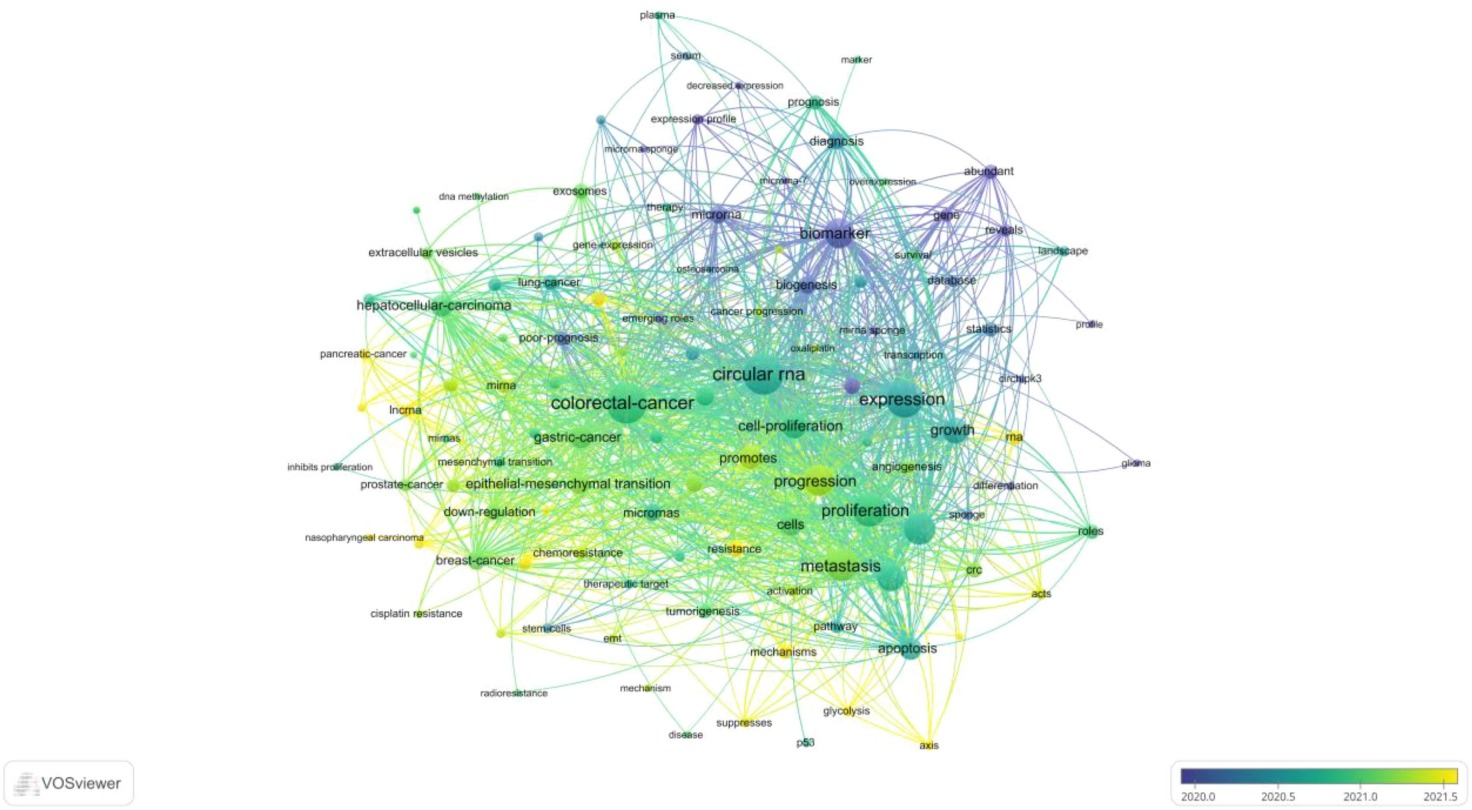
CRC on CircRNA research keywords for the VOSviewer visual network: visual mapping of keyword times. Distribution based on mean time of occurrence. The color of the circle represents the year of occurrence. Yellow color indicates most recent occurrence and purple color indicates earlier occurrence.

Keyword analysis, a common method for investigating popular research directions and areas, was conducted using VOSviewer to analyze all keywords with more than 8 occurrences. After eliminating redundant synonyms and meaningless words, 115 keywords were identified and classified into 5 categories: tumor research, malignant cell progression, biomarkers, diagnostic/prognostic related, clinical research on disease occurrence, and molecular biology-related research (as shown in **Figure 8**). The results of the keyword aggregation analysis yielded 5 research directions, each represented by different color blocks. For example, red clusters represent keywords such as CRC, gastric cancer, hepatocellular carcinoma, breast cancer, prostate cancer, nasopharyngeal carcinoma, etc. The blue cluster covers keywords related to proliferation, progression, expression, metastasis, growth, cells, apoptosis, etc. The green cluster includes biomarker, biogenesis, database, gene, sponge, survival, etc. The yellow cluster represents keywords related to diagnosis, prognosis, marker, therapy, serum, etc. The least frequent purple cluster consists of keywords such as p53, disease, tumorigenesis, mechanism, etc. **Figure 9** represents the temporal overlay plot of high-frequency keywords (n ≥ 8). It is evident that the mechanisms of tumorigenesis and development, as well as disease treatment, are emerging fields shown in yellow tones, indicating research hotspots in recent years. The top 10 most common keywords related to circRNA in CRC are listed in **Table 6**. CRC (338 occurrences, total linkage intensity 2059) is the most frequent keyword, followed by circular RNA (334 occurrences, total linkage intensity 1794), expression (289 occurrences, total linkage intensity 1565), and proliferation (249 occurrences, total linkage intensity 1318). Studies related to circRNAs in CRC primarily focus on improving treatment efficacy and reducing CRC-related mortality. Additionally, circRNAs can be used as potential biomarkers for cancer diagnosis and treatment.

**Table 6:** Top 10 keywords for number of studies related to CircRNA in CRC.

Keyword	Occurrences	Total link strength
colorectal-cancer	338	2059
circular rna	334	1794
expression	289	1565
proliferation	249	1318
metastasis	220	1228
invasion	208	1252
progression	192	1082
biomarker	173	963
migration	159	1010
growth	130	714

### 3.9 Publication keyword clustering and strongest keyword burst analysis

**Figure 10:**
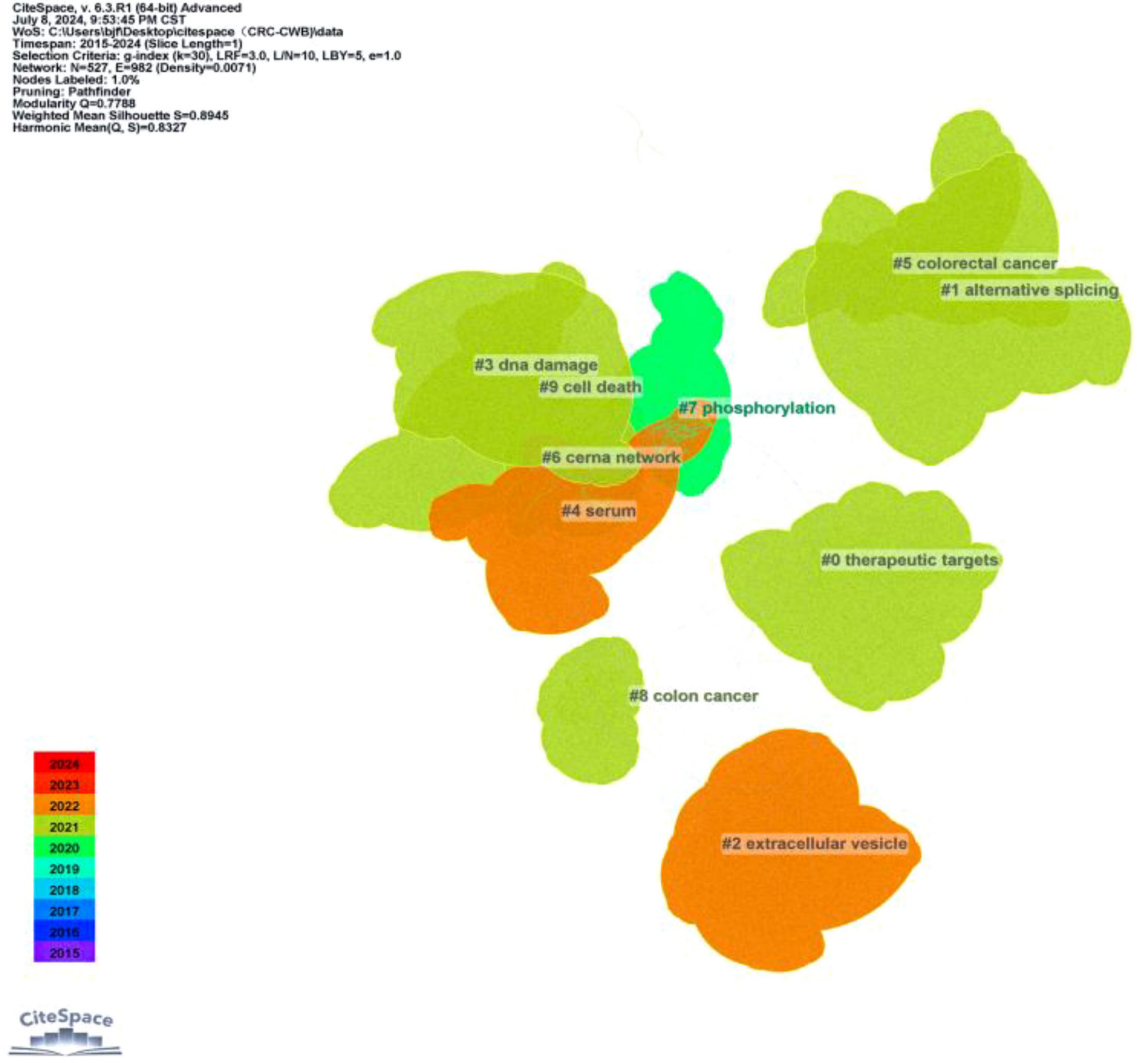
CiteSpace-based visualisation of co-occurrence networks for keyword class clustering in studies related to CircRNA in CRC.

**Figure 11:**
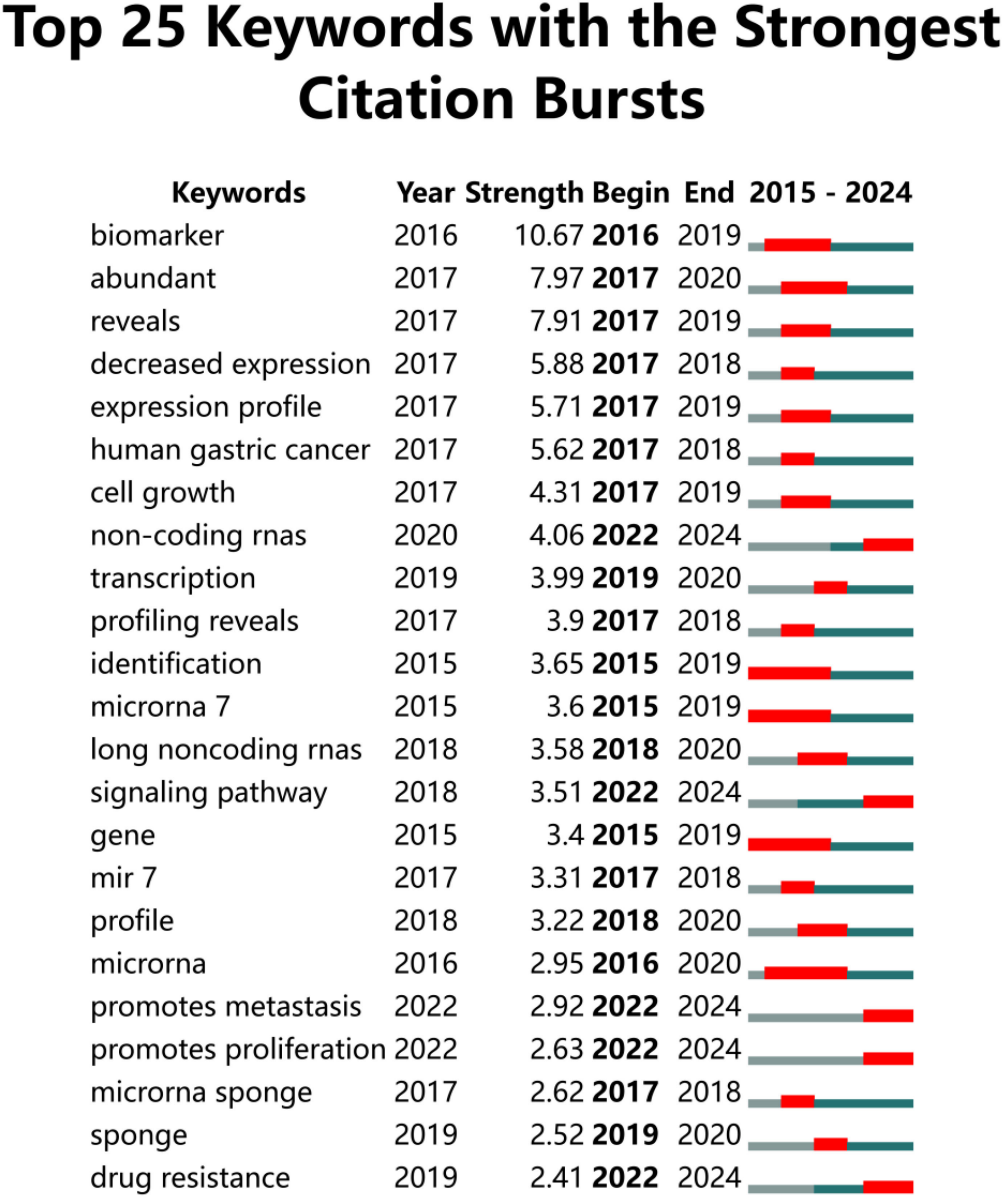
The top 30 keywords with the strongest CiteSpace-based citation bursts from 2015 to 2023. “Strength” indicates the strength of a keyword’s citation burst, with higher values indicating a higher frequency of occurrence during that time period. Light grey lines indicate less frequent time periods, lime green lines indicate more frequent time periods, and red markers indicate time periods when the keyword was at its most frequent.

A co-occurrence network visualization graph was constructed using CiteSpace for keywords. By combining proximate and similar keywords and adjusting the g-index from k = 25 to k = 30, a clearer picture of keyword distribution was obtained. The clustering of keywords is shown in **Figure 10**, with 10 clusters (Q = 0.7788, S = 0.8945), including #0 therapeutic targets, #1 alternative splicing, #2 extracellular vesicle, #3 DNA damage, #4 serum, #5 CRC, #6 cerna network, #7 phosphorylation, #8 colon cancer, and #9 cell death. **Figure 11** highlights the top 25 keywords with the strongest citation bursts, with ‘biomarker’ starting in 2016 and continuing through 2019, having the highest burst intensity of 10.67. This is followed by ‘abundance’ (2016-2020, intensity 7.97), and ‘reveals’ (2017-2019, intensity 7.91). This indicates that researchers initially focused more on circRNA as a new generation biomarker for CRC. Additionally, it was evident that non-coding RNAs (2020-2024, intensity 4.06), signaling pathways (2018-2024, intensity 3.51), promotion of metastasis (2022-2024, intensity 2.92), promotion of proliferation (2022-2024, intensity 2.63), and drug resistance (2019-2024, intensity 2.41) have also gained attention. Although the bursts are not as intense, they have continued until 2024. In short, circRNA, as a type of non-coding RNA, is further explored in terms of signaling pathways, promotion of cancer tissue proliferation and metastasis, and targeted drug resistance pathways. It is a hotspot of CRC research in the future.

## 4 Discussions

### 4.1 General information

Literature on circRNAs in CRC has been analyzed through bibliographic and visual analysis over the past 9 years, generating tables and images to reveal research advances, hotspots, and emerging trends. Research productivity in this field has increased due to the ongoing global investigation into circRNA biological functions and the rising burden of CRC, as researchers seek a deeper understanding of their relationship. The trend of this research hotspot has been rising annually, peaking in 2022. Along with dietary and lifestyle habits, the incidence of CRC varies significantly across different regions and countries. This also results in differences in the frequency and depth of CRC research in each region or country, leading to variations in the number of publications. As shown in **Table 1**, the number of papers and citations from China far exceeds those from other countries, and the top ten institutions are all located in China (e.g., **Table 3**), which suggests that CRC has a high incidence in China, and that there is a regional aspect to CRC, and that studying this area has a significant impact on the progress of cancer-related research in China. Despite China’s high number of papers and citations, the total intensity of linkages with other countries is not high, which may limit China’s regional research in CRC due to the fact that CRC morbidity and mortality have been on an upward trend in many low- and middle-income countries, whereas stabilising or decreasing trends tend to occur in highly developed countries, with regional variability of the disease. Addressing the challenge of CRC globally will require multifaceted efforts, including strengthening international cooperation, increasing public health awareness, improving healthcare infrastructure, and promoting effective screening and treatment strategies. Only in this way can the morbidity and mortality of CRC be reduced globally and human health and well-being enhanced. It is therefore important to strengthen cooperation with domestic and foreign institutions. Typically high GDP countries usually have greater research investments (funding, infrastructure, personnel, etc.), which in itself may lead to a higher absolute number of publications. However, this economic bias is not absolute, as shown by this paper’s comparison of the relationship between the number of original publications and GDP content. *Frontiers In Oncology*, *Molecular Cancer*, and *Oncotargets and Therapy* are among the top ten most productive journals, offering a rich source of high-quality literature for scholars seeking the latest knowledge on circRNA frontiers and hotspots in CRC research. Among the authors, Hushmandi Kiavash is the most productive, with the highest citation and linkage strength index, highlighting the relationship between non-coding RNAs and cancer progression and treatment mechanisms, as well as the potential application of non-coding RNAs as biomarkers and therapeutic targets in gastrointestinal tumors (60, 61). The incidence of CRC has been rising with economic development and changes in people’s living and dietary habits (22), and the burden of CRC has been increasing, with more young people being diagnosed with advanced disease at the diagnostic stage (2). Therefore, further early diagnosis, intervention, and improved treatment are imperative. Currently, colonoscopy has high sensitivity for CRC and is effective for early detection and diagnosis (23). However, it has disadvantages, such as being invasive and expensive, and the applicable age for screening is controversial (24). Studies have shown that circRNAs play a role in many aspects of gene expression, from regulating transcription in the nucleus to translation in the cytoplasm (25). Therefore, there is a need to utilize circRNAs as a non-invasive, low-cost, and reliable screening tool. The correlation of circRNAs in the histocytes of patients with CRC has undeniable potential for early diagnosis (26).

### 4.2 The biological function of circRNA in CRC

Based on the statistics of keywords, article heat, a total of several research hotspots in this field were summarized. circRNA’s role as an emerging biomarker for CRC is a hot topic (13), after several basic research trials, the circRNA expression levels in CRC patients varied, circRHOBTB3 has low expression in tumour tissues, and tumour size is in its negative correlation (27); circPACRGL is highly expressed in CRC cells, and through the axis of signalling pathway, it was found that the inhibition of its expression can reduce the proliferation and metastasis of cancer cells (28); CircLPAR1 has significantly decreased expression in plasma of colorectal tumour patients, which is positively correlated with the survival curve of the patients (29); CircAFF2 is able to increase the radiosensitivity of CRC patients, and beneficial to pre-operative radiotherapy sensitivity of CRC patients, which is beneficial for preoperative neoadjuvant radiotherapy (30); CircATG4B is overexpressed in oxaliplatin-resistant CRC cells, which can induce autophagy to improve drug resistance (31); CircMYH9, which is abundantly present in blood samples of CRC patients, can stimulate the production of immune antigens by CD8+T, providing a new therapeutic pathway (32); CircPPFIA1-L and -S have inhibitory effects on metastatic properties and are significantly down-regulated in histiocytes of liver metastatic CRC, which can be used as target genes to inhibit tumourigenesis (33). All these features reflect the biological functions of circRNAs, which can make them potential markers for the diagnosis and prognosis of CRC. However, the specificity of circRNA expression in CRC is limited, and further research is still needed regarding technical issues and biological knowledge gaps to incorporate circRNA into the clinic for helping cancer patients (8).

### 4.3 The potential of circRNA as a biomarker

CRC is one of the most common malignant tumors of the digestive tract. Research indicates that CRC originates from an abnormal crypt, gradually forming an adenomatous polyp, and over several years, transforms into sporadic CRC, a traditional process accounting for about 80% of CRC cases (34). This highly malignant tumor also has a complex pathogenesis, based on chromosomal instability (CIN), microsatellite instability (MSI), and CpG island methylation phenotype (CIMP) (35). Factors such as environmental and genetic influences are also crucial. In CRC, gene expression regulation by circRNA is a significant mechanism, primarily exerting its biological function in the cytoplasm by selectively regulating processes like gene transcription and splicing (36). Some studies have shown that circRNA is down-regulated in mutant KRAS cells, qRT-PCR analysis revealed that down-regulation of circRNA can cause changes in the corresponding host genes (37). CircRNAs can influence the expression of target genes by competitively binding to microRNAs or acting as miRNA sponges, the most widely studied mechanism of circRNAs in CRC progression (38). For instance, CircHIPK3 is significantly up-regulated in CRC tissues and cells, functioning as a miR-7 sponge (tumor suppressor), and its overexpression can effectively reverse miR-7’s function and promote cancer (39). CircRNAs can also interact with RNA-binding proteins, causing changes in protein activity (40). Chen et al. found that CircNSUN2 is mainly upregulated in liver metastatic CRC tissues, with its expression being abundantly stable and invasive. CircNSUN2 expression is enhanced by forming a CircNSUN2/IGF2BP2/HMGA2 RNA-protein ternary complex that stabilizes HMGA2 mRNA, serving as a prognostic indicator for patients with liver metastatic CRC (41). Additionally, many signaling pathways in the human body regulate cellular processes and autoimmune conditions. Increasing evidence suggests that circRNAs can affect CRC development by regulating signaling pathways (42). For example, cir-ITCH is highly expressed in most CRC tissues, interacting with various miRNAs. Experiments have demonstrated that cir-ITCH can participate in regulating the Wnt/β-catenin signaling pathway *in vivo*, inhibiting signal transduction and suppressing oncogenes (43).

### 4.4 The therapeutic application and future research directions of circRNA in gastrointestinal tumors

Additionally, extensive research on circRNAs has led researchers to further explore their clinical application as cancer therapeutic targets (44). Aberrant expression of circRNAs is observed not only in CRC tissues and cells but also plays a crucial role in the pathogenesis of other gastrointestinal cancers (45). It was found that CircBCAR3 is up-regulated in esophageal cancer tissues and cells, with increased expression under hypoxia. Experimentally, knockdown of CircBCAR3 suppressed the levels of waveform protein, N-calmodulin. Potential miRNAs bound by CircBCAR3 were predicted from databases, establishing a signaling axis and targeting CircBCAR3 as a potential therapeutic option (46). Wang et al. found that circURI1 functions as a miRNA sponge, binding proteins to regulate metastatic factors, thus inhibiting metastasis of cancerous tissues. The study indicated that CircURI1 is one of the few selectively spliced circular RNAs involved in cancer metastasis, presenting a new research breakthrough (47). Summarizing the reports, the function and expression of circRNAs can be used as potential biomarkers for gastrointestinal cancers. Whether based on gene regulation, the competing endogenous RNA (ceRNA) hypothesis, or binding protein interactions, the expression of circRNAs can be found in human tissues, cells, or body fluids, providing objective evidence to assess their function. Starting with circRNAs as new diagnostic and therapeutic strategies, further studies in circRNA and cancer are needed to fully characterize their role in cancer progression or suppression (48).

### 4.5 Evolution of hot spots and research frontiers

Analyses of keyword highlights, popular articles, and keyword timeline maps reflect the development process and frontiers of a field. The initial 2015 study on circRNA and CRC focused on exploring the diverse functions and mechanisms of circRNAs (49). It investigated the biological role of cir-ITCH in CRC by regulating the Wnt/β-catenin signaling pathway (43) and hsa_circ_001988 in CRC expression in tissue cells to examine its clinical significance (50). From 2016 to 2020, researchers explored the function of circRNAs by examining differentially expressed circRNAs in cancer and normal tissues, focusing on signal transduction pathways, targeting or sponging miRNAs, and hypothesizing competing endogenous RNA (ceRNA) networks. These studies aimed to understand the impact of circRNAs on CRC (40, 51–54). As research on circRNAs peaked in 2022, studies confirmed that circRNAs are closely associated with angiogenesis in CRC (55), that vasculature is critical for tumor growth, and that circRNAs are widely present in eukaryotic cells with disease specificity, making them potential biomarkers for CRC diagnosis (56). Further investigations into the effects of circRNAs on proliferation, apoptosis, migration, invasion, and angiogenesis of tumor tissues were also highlighted (57–59). In recent years, keywords such as ‘metastasis’, ‘proliferation’, and ‘drug resistance’ have received significant attention, highlighting the future direction of this field. The focus in this field has shifted from studying circRNA biological characteristics to regulating circRNA molecular mechanisms, indicating that scholars are increasingly viewing circRNAs as potential biomarkers for the diagnosis, prognosis, and treatment of CRC. Therefore, addressing the technical issues to advance the field and facilitate its clinical application is essential.

### 4.6 Prospects and limitations

To the best of our knowledge, this is the first bibliometric statistical and visualization analysis of the current status and trends of circRNA research in the field of colon cancer. This study covers 9 years of research, enabling us to visualize hotspots, observe global research trends, and promote academic development in this field. WOSCC is widely regarded as the most reliable database for bibliometric analyses, containing the most influential academic journals globally. Therefore, we believe our findings effectively reflect the overall status and trends of the discipline. However, our study has some limitations: first, we only retrieved data from the WOSCC core database, which enhances the quality and standard of included publications but may overlook influential publications in other databases, resulting in a lack of breadth in the data for analysis. Second, our search was limited to articles published in English from early 2015 to late 2023, excluding articles that were not accepted or had errors, potentially presenting an incomplete picture of long-term research trends. Nonetheless, this study sufficiently highlights the research hotspots and primary concerns in this field globally.

## 5 Discussion

This study represents the first bibliometric analysis of circRNAs in CRC, outlining the development and evolution of publications in the field over an extended period. Our findings highlight the most influential countries/regions, prestigious institutions, journals, and authors in this field worldwide, indicating the most impactful publications. We hope that our findings will offer valuable insights for future research directions in the diagnosis and treatment of CRC. Currently, further studies are needed to elucidate the specific sensitivity of circRNAs in CRC, identify therapeutic targets and treatment options, and promote the translation of research findings into clinical practice.

## Data Availability

The original contributions presented in the study are included in the article/supplementary material. Further inquiries can be directed to the corresponding authors.
